# Patterning the Axes: A Lesson from the Root

**DOI:** 10.3390/plants8010008

**Published:** 2018-12-31

**Authors:** Riccardo Di Mambro, Sabrina Sabatini, Raffaele Dello Ioio

**Affiliations:** 1 Department of Biology, University of Pisa, via L. Ghini, 13-56126 Pisa, Italy; riccardo.dimambro@unipi.it; 2 Dipartimento di Biologia e Biotecnologie, Laboratory of Functional Genomics and Proteomics of Model Systems, Università di Roma “Sapienza”, via dei Sardi, 70-00185 Rome, Italy; sabrina.sabatini@uniroma1.it

**Keywords:** *Arabidopsis*, root, stem cells, root development, differentiation, ground tissue, radial patterning, proximodistal patterning

## Abstract

How the body plan is established and maintained in multicellular organisms is a central question in developmental biology. Thanks to its simple and symmetric structure, the root represents a powerful tool to study the molecular mechanisms underlying the establishment and maintenance of developmental axes. Plant roots show two main axes along which cells pass through different developmental stages and acquire different fates: the root proximodistal axis spans longitudinally from the hypocotyl junction (proximal) to the root tip (distal), whereas the radial axis spans transversely from the vasculature tissue (centre) to the epidermis (outer). Both axes are generated by stereotypical divisions occurring during embryogenesis and are maintained post-embryonically. Here, we review the latest scientific advances on how the correct formation of root proximodistal and radial axes is achieved.

## 1. Introduction

**Figure 1:**
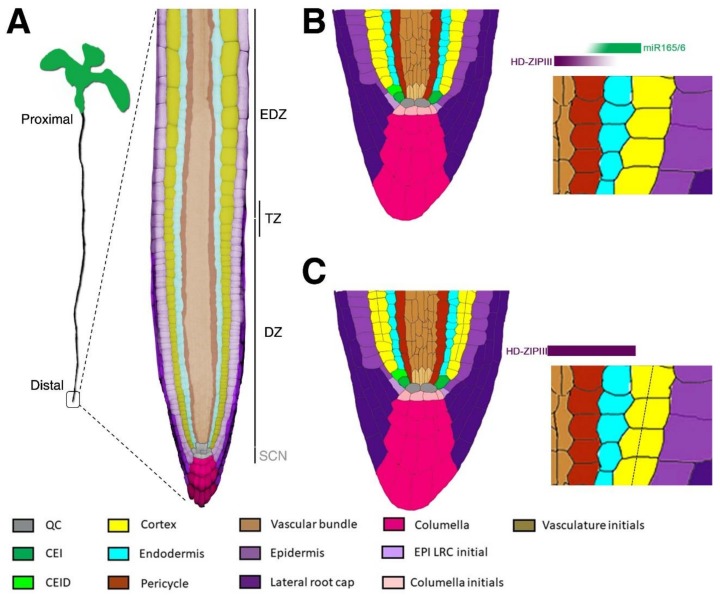
*Arabidopsis* root structure. (**A**) Representation of an *Arabidopsis* seedling where the proximodistal axis is indicated. In the blow up, a representation of the *Arabidopsis* root apex is shown where false colours highlight the different tissues. Root zonation: stem cell niche, SCN; division zone, DZ; elongation/differentiation zone, EDZ; transition zone, TZ. (**B**) Cartoon reporting the longitudinal section of a wild type (Wt) *Arabidopsis* root stem cell niche. Different colours represent root tissues and initials, as indicated in the legend. The blow up highlights the typical ground tissue (GT) architecture (one layer of endodermis and one layer of cortex) resulting from the opposite graded distribution of miR165/6 and Class III Homeodomain Leucine Zipper (HD-ZIPIII) (triangle shapes above blow up). In particular, miR165/6 (green) presents low expression in the vascular bundle and high expression in the endodermis, constraining HD-ZIPIII expression. As a result, HD-ZIPIII (red) present high expression in the vascular bundle and low expression in the endodermis. (**C**) Cartoon reporting the longitudinal section of an *Arabidopsis* stem cell niche lacking miR165/6 expression. The blow up highlights the HD-ZIPIII expanded expression in the whole ground tissue (GT). This results in the formation of an extra layer of the cortical tissue (dashed line). QC, quiescent centre; CEI, cortex and endodermis initial; CEID, cortex and endodermis initial daughter cell; EPI, epidermis; LRC, lateral root cap.

One of the most intriguing questions in developmental biology is how the body plan is established. To answer this question, for decades scientists have focused on the formation of developmental axes, utilizing different model systems. Most of our knowledge on axes formation derives from studies on vertebrate limb development [1,2]. However, these systems present several limitations due to their complex structure that limits analysis at a single cell resolution. On the contrary, plant roots display a simple and organized structure, where cell lineages are easily distinguishable by shape and position [3,4]. Furthermore, due to the presence of the cell wall, plant cells do not migrate; hence, cell fate and identity can be easily followed during different stages of organ development [3,4,5]. For these reasons, roots represent a powerful tool to study the molecular mechanisms on how developmental axes are established and maintained. Roots can be represented as a series of concentric cylinders, where epidermis is the outermost tissue while the vasculature bundles lie in the centre [4] (Figure 1). Roots display two main developmental axes: the proximodistal axis, extending longitudinally from the root–shoot junction (proximal) to the root apex (distal); the radial axis, spreading transversally from the vasculature bundles to the epidermis [4] (Figure 1). Like other animal model systems, root axes are established during embryogenesis and maintained post-embryonically by the activity of meristems [3,4,5,6]. Meristems are localized structures that sustain post embryonic indeterminate plant organ growth due to the activity of stem cell niches (SCNs) [3,4,5]. In the *Arabidopsis* root meristem, there are five sets of stem cells (initials) that give rise to all root tissues: epidermis and lateral root cap initials (EPI LRC STEM CELLS), cortex and endodermis initial (CEI), pericycle initials, vasculature initials and distally columella initials (Figure 1) [3,4]. These sets of stem cells surround the QC (Quiescent Center) which maintains, by contacting them, their stem cell identity (Figure 1) [7,8]. The stem cells divide asymmetrically and anticlinally generating daughter cells (Figure 1) that generates both the proximodistal and radial axes through stereotypical cell divisions. Along the proximodistal axis, the stem cell daughters divide anticlinally a fixed number of times, generating the division zone of the meristem. In the proximal area of the meristem, those cells cease to divide when they reach a boundary called the transition zone (TZ). Here they start to elongate and differentiate, generating the elongation/differentiation zone [3,5,9,10] (Figure 1). In this zone, cells acquire characteristic differentiation features such as root hairs for the epidermis or tracheids for the vascular cells [11,12]. The position of the TZ is fundamental for proximodistal axis specification, as it marks the boundary between undifferentiated and differentiated cells [9,13].

Radially, most of the stem cells daughters divide periclinally, giving rise to two tissues with different identities. For example, cortex and endodermis are derived from the periclinal division of the daughter of the cortex and endodermis initial (CEI), whereas epidermis and lateral root cap originate from the EPI LRC initial [14,15,16]. The control of the asymmetric divisions occurring in the stem cell daughters is key for the correct patterning of the radial axis. Indeed, alteration of the position and timing of those divisions causes the formation of aberrant body plan and shape (Figure 1). 

Thanks to the generation of new tools and the improvement of molecular methodologies, several molecular mechanisms underlying the establishment and maintenance of the proximodistal and radial are in the process of being discovered and fully comprehended. In this review, we report the current view on how these two axes are patterned.

## 2. Root Radial Axis 

The root radial axis organization depends on the coordinated activity of periclinal divisions of the stem cell daughters. One of the most studied mechanisms patterning the radial axis is the one controlling the formation of the cortex and the endodermis root tissues. These tissues originate from a single stem cell (CEI) that firstly divides anticlinally, thereby generating a daughter cell (CEID). This cell divides periclinally, generating the cortex and the endodermis that together are called Ground Tissue (GT). GT specification starts in the embryo when a periclinal division at early globular embryonic stage separates the pro-vasculature tissues from the GT precursor cell. Only later, at the heart embryonic stage, a pro-GT division leads to the specification of the cortex and the endodermis [6]. It was recently shown that the establishment of the pro-GT at early embryonic stages depends on the plant hormone auxin. A maximum level of auxin activity driven by the auxin responsive factor MONOPTEROS/AUXIN RESPONSIVE FACTOR 5 (MP/ARF5) in the GT precursor cells is required for GT formation [17]. Indeed, *mp* null mutants display impaired GT establishment [17]. 

**Figure 2:**
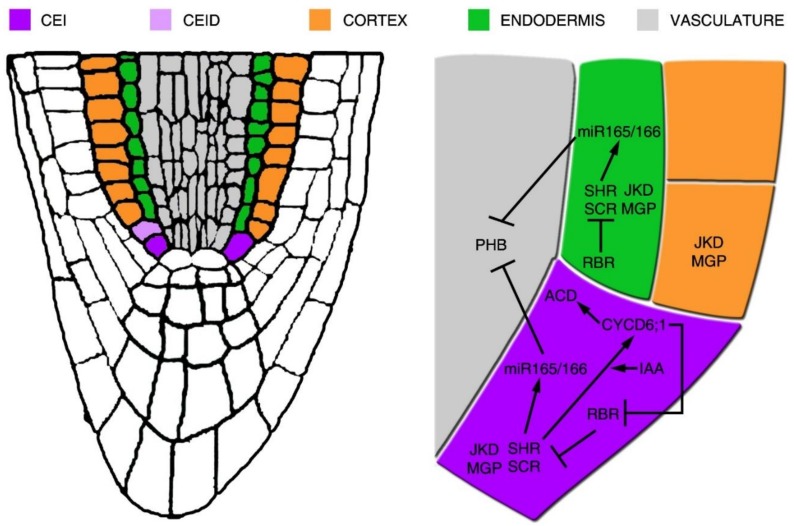
Schematic representation of the gene regulatory network acting for the cortex and endodermis initial periclinal division. On the left, representation is provided of the *Arabidopsis* root tip, where the cortex and endodermis initial (CEI) and its daughter cell (CEID), cortex, endodermis and vascular tissues are depicted in colour. In the blow up, the gene regulatory network supporting the CEI asymmetric cell division (ACD) is shown. In the CEI, the SHR/SCR complex sustains the expression of SCR and promotes the expressions of CYCD1;6 and of JKD and MGP. CYCD6;1 expression is also sustained by high levels of auxin (IAA) in the CEI. CYCD6;1 represses RBR activity, which in turn regulates negatively the ACD by a direct repression of SCR activity. SHR/SCR complex also promotes the expression of miR165/6, thus restricting PHB expression in the vascular tissue.

**Figure 3:**
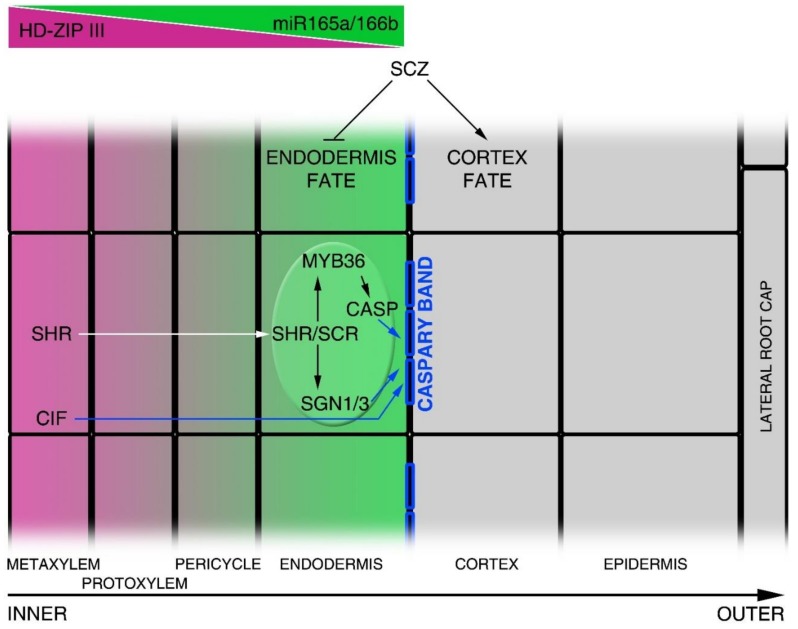
Image showing the molecular mechanisms controlling radial axis patterning. The figure shows a half radial section of the *Arabidopsis* root. Each square file corresponds to a different tissue layer, where the central file (inner) corresponds to the metaxylem and the outer file to the lateral root cap, as indicated in the scheme. Class III Homeodomain Leucine Zipper III (HD-ZIPIII) and miR165a/6b (opposite gradients) are indicated in purple and green, respectively. Blue squares on endodermal cells represent Casparian strips. White arrow indicates SHR protein movement from the vascular tissue into endodermal cell nucleus. Blue arrows indicate the CIF-, CASP- and SGN1-dependent regulation of Caspary band formation. SCZ promotes the cortical identity supporting tissue fate separation.

Two GRAS family transcription factors, SHORTROOT (SHR) and SCARECROW (SCR), are involved in the formation of the cortex and endodermis layers, as they are necessary and sufficient to promote the CEID periclinal division [18,19,20,21]. SHR is a mobile transcription factor expressed in the vasculature. SHR moves toward the CEID, CEI and endodermis via plasmodesmata, where it is sequestered into the nucleus [22,23] (Figure 2 and Figure 3). SHR movements restriction is fundamental for GT patterning, as overexpression of SHR results in additional GT layer formation [24,25]. In the vasculature, SHR is maintained mostly in the cytoplasm by the activity of SCARECROW-LIKE23 (SCL23) [26]. In the CEID, SHR forms a molecular complex with SCR and it is sequestered in the nucleus by the activity of SCR. In the nucleus, SHR/SCR complex sustains the expression of *SCR* itself and induces the expression of *INDETERMINATE DOMAIN C2H2 zinc finger* (*BIRD*) transcription factors such as *JACKDAW* (*JKD*), *NUTCRACKER* (*NUC*) and *MAGPIE* (*MGP*) [21,27,28,29,30,31]. BIRD proteins physically interact with the SHR/SCR complex, restricting SHR movements to the stele [27,29,32]. SHR/SCR complex promotes the expression of the cell cycle regulator *CYCLIND6* (*CYCD6;1*) in the CEID, inducing here a periclinal division [33,34]. Via a combination of mathematical modelling and wet biology, it has been proposed that the SHR/SCR/CYCD6;1 module, together with the cell cycle inhibitor RETINOBLASTOMA-RELATED (RBR) protein, acts via a bistable circuit to regulate the CEID asymmetric division [33,34,35]. In the CEI, the CYCD6;1 together with the CDKB1;1 (CYCLIN DEPENDENT KINASE 1;1) or CDKB1;2 induces the phosphorylation of RBR, reducing its activity in the CEID, thus promoting the periclinal division [34]. Auxin is a key factor for the promotion of this periclinal division. Indeed, an auxin maximum in the CEI promotes *CYCD6;1* expression [34]. On the contrary, RBR was shown to directly interact with SCR, reducing its transcriptional activator activity in the endodermis [36]. The RBR and SCR interaction, together with the activity of the RBR regulator CYCD6;1, limits the asymmetric cell division in the SCN, thus allowing the formation of the endodermal and cortical layers (Figure 2). Recently, a sophisticated molecular mechanism was proposed for a SHR/SCR-dependent switching on of the CYCD6;1 involving the RNA POLYMERASE II cofactor Mediator. Depending on the SHR concentration, SCR interacts with the subunit 31 of the Mediator to promote *CYCD6;1* expression [37].

Once CEID divides, several factors coordinate the formation of the cortical and endodermal layers. SHR also promotes endodermal fate, as suggested by the loss of endodermis identity in *shr* mutants [18,28,38,39]. It was shown that BIRD proteins, other than regulating SHR movements, play a key role in determining cortical identity, as multiple mutant combinations of BIRD members show GT with no cortical identity [32]. Therefore, the combined activity of SHR, SCR and BIRD proteins is necessary to pattern the GT. Interestingly SHR and SCR are involved only in the maintenance of GT and not in its establishment. Once MP initiates the ground tissue lineage, it acts upstream of the SHR/SCR module, controlling ground tissue patterning and maintenance.

SCHIZORIZA (SCZ), a member of the Heat Shock Transcription Factor family, is also involved in GT patterning and its activity depends on SHR and SCR [40,41,42]. Interestingly, SCZ is expressed in all root tissues except for the lateral root cap. It was shown that SCZ, together with JKD, MGP, and NUC proteins, promotes cortical identity (Figure 2 and Figure 3) [32]. It must be pointed out that *scz* mutants present additional tissue layers with mixed cortical, endodermal and epidermal identities, suggesting a role for this gene in tissue fate separation [40,41,42]. The analysis of SCZ target genes will help to establish an understanding of how SCZ patterns the radial axis. 

Besides organizing the GT, SHR/SCR complex is involved in vasculature patterning. *Arabidopsis* root vasculature consists of an inner xylem bundle (metaxylem in the centre, protoxylem aside) with two juxtaposed phloematic bundles [4] (Figure 3). The formation and development of the metaxylem depends on the redundant activity of the Class III Homeodomain Leucine Zipper (HD-ZIPIII) members, a family of five transcription factors targeted by microRNA 165/6 (miR165/6). SHR/SCR promotes in the endodermis the expression of miR165/6 that, moving toward the stele via plasmodesmata, generates an opposite gradient of the HD-ZIPIII proteins, with a maximum in the metaxylem and a minimum in the endodermis [22,43,44] (Figure 1 and Figure 3). The formation of a radial gradient of HD-ZIPIIIs is sufficient to pattern the xylem fate specification, as high HD-ZPIIIs levels promote metaxylem formation, whereas low ones promote protoxylem [43,44] (Figure 3). In the stele, HD-ZIPIIIs control the biosynthesis and activity of the phytohormone cytokinin, which in turn regulates auxin distribution and signalling [44,45]. This finely regulated mechanism is sufficient to pattern the stele.

It was recently shown that miR165/6 distribution is not only crucial for vasculature development but also for GT patterning [46,47] (Figure 1 and Figure 2). A mir165/6-dependent minimum of HD-ZIPIIIs in the CEI/CEID and endodermis is required to restrict the number of cortical layers, as miR165/6-insensitive HD-ZIPIII mutants show additional cortical layers [47]. HD-ZIPIIIs expression in the GT results in ectopic *CYCD6;1* activation, prompting additional GT divisions. Intriguingly, the HD-ZIPIII member PHABULOSA (PHB) indirectly sustains *CYCD6;1* expression in a SHR-independent manner, but how PHB triggers periclinal divisions is still not known [46,47,48]. It was recently shown that PHB directly targets MP to pattern the vasculature tissue [49]. Nevertheless, whether PHB/MP circuit is important for GT establishment is not known.

It was recently shown that SHR, together with SCR, also specifies endodermis differentiation. Functional endodermis is characterized by Casparian strips, lignified structures deposited on the radial and transverse side of the endodermal cell wall [24]. SHR directs the formation of the Casparian strips by inducing the MYB DOMAIN transcription factor *MYB36* and the receptor-like kinase *SGN1* and *SGN3* [50,51] (Figure 3). In endodermal cells, MYB36 induces the expression of the transmembrane proteins CASPARIAN STRIP MEMBRANE DOMAIN PROTEIN (CASP) [52,53], which are involved in the recruitment of lignin synthesis enzymes on the plasma-membrane. SGN1/3 position CASP proteins on the plasma membrane (Figure 3). Nonetheless, SHR promotes the formation of a non-functional Caspary band and it requires the activity of vasculature-deriving small peptides, *CASPARIAN STRIP INTEGRITY FACTOR* (*CIF*), to generate a functional strip [50,51] (Figure 3). Hence, SHR and SCR constitute an important module to control endodermis differentiation.

## 3. Root Proximodistal Axis 

**Figure 4:**
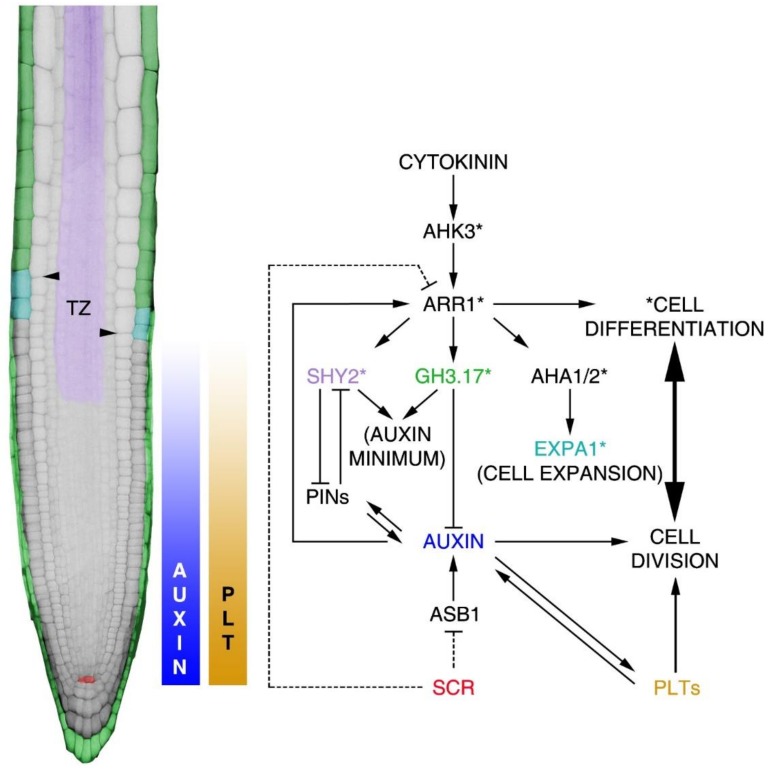
Molecular mechanisms leading to the root proximodistal axis patterning. On the left, representation of the *Arabidopsis* root. False colours indicate the activity domain of genes involved in the regulation of the equilibrium between cell division and cell differentiation processes established in the root. Arrowheads indicate the position of the TZ. Auxin and PLT-graded distributions are indicated in the piecewise colour bar, where the maximum is in the SCN and the minimum at the TZ. The molecular mechanisms acting in the control of meristem activity are indicated in the diagram. The QC SCR domain is represented by red; in green, the root cap and differentiated epidermis GH3.17 domain; in purple, the vascular bundles SHY2 domain; and in light blue, the first elongating epidermal cells EXPA1 domain. Asterisks represent genes involved in the regulation of the cell differentiation process mediated by cytokinin activity. Dashed lines indicate SCR-dependent indirect regulation of ASB1 and ARR1.

Different from the radial axis, where most of the cells show different identities but similar developmental stages, along the proximodistal axis cells display different stages of development. Positioning of the TZ plays a key role for patterning the proximodistal axis, as the TZ separates proliferating meristematic cells from the elongated ones [54] (Figure 1 and Figure 4). The position of the TZ depends on the dynamic equilibrium between cell division and cell differentiation; alterations of this equilibrium cause the TZ position to shift toward the distal or the proximal area of the root, thus varying the proximodistal zonation. 

Auxin plays a pivotal role in establishing the root proximodistal axis, acting as a local morphogen [55,56]. Already at the globular stage of embryogenesis, a maximum of auxin in the basal pole of the embryo determines the position of the SCN [57]. This auxin maximum is controlled by the activity of the auxin polar transport efflux facilitators PIN FORMED (PINs) that distribute this hormone [58,59,60]. Auxin signalling is necessary for the formation of the SCN [55,61]. Interestingly, *mp* loss of function mutants or gain of function mutants of its repressor, the AUX/IAA auxin signalling repressor BODENLOS (BDL), display no root formation [61,62,63]. Together with auxin, four AP2 transcription factors, PLETHORA 1,2,3 and 4 (PLT1,2,3 and 4), control stem cell activity and root growth from embryogenesis onwards [64,65]. Multiple combinations of the loss of function mutants *plt1,2,3,4* show no root SCN formation, whereas constitutive expressions of *PLT* genes induces shoot homeotic transformation into root [65]. The GATA transcription factor HANABA TARANU/MONOPOLE (HAN) forms the boundary between embryonic apical and basal pole, confining PLT expression and the auxin maximum to the root precursors domain [66]. PLTs also play also an active role in the repression of the apical pole identity. Indeed, PLT, together with miR165/6, represses the apical embryonic SCN formation by restricting HD-ZIPIIIs expression [67,68]. Lack of this repression leads to the homeotic transformation of the root into shoot, suggesting a master role for PLT in determining the embryonic apical-basal axis [68]. It has been recently demonstrated that PLT regulates the expression of *HAN* and the synthesis of auxin via direct control of *YUCCA3*, a gene involved in auxin biosynthesis [69]. One possibility is that PLTs regulate the expression of genes involved in apical fate determination, such as HD-ZIPIII directly acting on *HAN* or on auxin synthesis. Future studies will clarify this point.

Post-embryonically, PLTs and auxin are required to maintain SCN activity in the root, forming a gradient with a maximum in this zone (Figure 4) [55,64,65,70]. Ectopic inductions of auxin or PLTs maximum in the meristem convert other cell types in stem cells, underlying the importance of these maxima for stem cell specification. Post-embryonically, PLTs mRNAs and auxin are distributed in a gradient along the meristematic proximodistal axis [70]. PLTs and auxin gradients are strictly interconnected. Indeed, auxin promotes PLTs expression, whereas PLTs regulate auxin distribution, controlling PINs expression and auxin biosynthesis [58,64,69]. Different concentrations of auxin or PLTs result in different developmental outputs, i.e., high PLTs and auxin levels are necessary for SCN specification, whereas minimum auxin and PLT levels are necessary to induce cell differentiation at the TZ [55,64,65,70,71] (Figure 4). Recent studies have shown that the PLTs gradient along the proximodistal axis is partially independent from auxin, while the capacity of these proteins to diffuse along this axis plays an important role [65,70].

PIN-dependent polar auxin transport is necessary to position the auxin maximum at the root distal part [55,59] and the manner in which an auxin minimum is positioned at the proximal TZ has recently been elucidated. Indeed, the role of the plant hormone cytokinin in shaping the auxin gradient has been revealed. To position this minimum cytokinin triggers a module that involves the cytokinin receptor AHK3 (*ARABIDOPSIS* HISTIDINE KINASE 3), the cytokinin-dependent transcription factor ARR1 (*ARABIDOPSIS* RESPONSE REGULATOR1), the auxin signalling repressor SHY2/IAA3 (SHORT HYPOCOTYL2/INDOLE-3-ACETIC ACID INDUCIBLE 3) and the auxin catabolic enzyme GH3.17 (GRETCHEN HAGEN 3.17) [54,71,72,73] (Figure 4). Cytokinin via AHK3 activates ARR1 directly inducing the expression of *SHY2* in the vasculature at the TZ. Here, SHY2 downregulates *PINs* expression, thus reducing the shoot to root auxin efflux and, hence, cell division activity; auxin instead induces proteasome-dependent SHY2 degradation, supporting PINs expression [54,72,73,74] (Figure 4). At the same time, cytokinin via ARR1 induces the *GH3.17* gene in the lateral root cap and epidermis [71], where it mediates auxin degradation (Figure 4). The coordinated regulation of both auxin signalling and catabolism localizes a developmental instructive auxin minimum that positions the TZ [71]. 

The coordination of SCN activity with cell differentiation process at the TZ is fundamental for proper proximodistal axis patterning. This spatial coordination is controlled by SCR and SHR, which repress cytokinin activity in the SCN, thus controlling auxin production [34,75,76]. In particular, SCR represses ARR1 expression in the QC, which in turn positively regulates the expression of the auxin biosynthesis gene *ASB1* (*ANTHRANILATE SYNTHASE BETA SUBUNIT 1*). Since ARR1 is induced by auxin at the TZ SCR, by regulating auxin biosynthesis in the QC, this controls stem cell division in the SCN and cell differentiation at the TZ [34].

Considering the key role of cytokinin in positioning the TZ, the regulation of cytokinin synthesis is fundamental for proper root patterning [13,24]. ISOPENTENYL TRANSFERASE (IPT) enzymes are key regulators of cytokinin synthesis [24]. In the meristem, the transcription factor PHB promotes cytokinin synthesis via direct induction of *IPT1* and *IPT7* [77]. The PHB-dependent cytokinin production is sufficient to activate the ARR1/SHY2 module, positioning the TZ. Intriguingly, ARR1 represses both PHB and its repressors miR165/6 expression, triggering a negative incoherent feedforward loop that finely tunes cytokinin levels and prevents meristem from differentiating [77]. 

Recently, it was demonstrated that cytokinin also promotes the transition of cells from the meristematic zone to the elongation zone, regulating apoplastic acidification and cell expansion. ARR1 directly regulates enzymes involved in cellular expansion such as the *α-expansin EXPANSIN1* (*EXPA1*), controlling cell wall loosening and the plasma membrane H^+^-ATPases (HA) 1 and 2 (AHA1 and AHA2) that transport protons (*H^+^*) out of the cell. Interestingly, *expa1* mutants show a shift of the TZ toward the root proximal zone without interfering with the final cell size, suggesting that EXPA1-dependent cell expansion is mostly controlling the timing of cell exit from the division zone more than the final cell size itself [78] (Figure 4).

## 4. Future Perspectives

In recent years we have increased our understanding of the mechanisms controlling the formation of both radial and proximodistal axes of the root apical meristem. It is interesting to notice that the molecular mechanisms patterning both the radial and the proximodistal axes involve the same main players (i.e., auxin, PHB and SHR/SCR). Future studies will elucidate how the mechanisms controlling the development of these two axes coordinate in order to generate a structured body plan. Moreover, the effectors of the master genes governing the zonation of both those axes are starting to be understood, but several players are still missing and will be discovered in the future. In this optic, with respect to the proximodistal axis, the lists of targets of ARR1 and PLTs were published. This knowledge will allow us to better understand how those genes are interconnected and how they coordinate to ensure continuous growth. 

In multicellular organisms, cell elongation is accompanied by endoreduplication, genome duplication in absence of mitosis [79,80]. Similarly, it was shown that cells at the TZ show enhanced the number of genome copies compared to their meristematic progenitors. Moreover, cytokinin is known to promote endoreduplication [81]. It will be interesting in the future to investigate the role of endoreduplication in patterning the root proximodistal axis. 

Most of the molecular mechanisms patterning the root axes were discovered in *Arabidopsis*. In recent years, several variations in those mechanisms were found to be the basis for interspecific variability in plants. 

Indeed, root axis structure are largely variable among species. For example, the root radial axis is extremely variable, as number and features of tissue layers is strictly dependent on the species [24,82,83]. One of the tissues along the radial axis that is most variable between species is the cortex [24]. For example, *Cardamine hirsuta*, a close relative of *Arabidopsis*, displays two cortex layers. It was recently shown that the second cortical layer of *Cardamine* emerges from the activity of a developmental domain absent in *Arabidopsis*, the cortex endodermis mixed identity tissue (CEM). Activity of HD-ZIPIII in this domain is crucial for the formation of the second cortical, as knockdown of these genes results in the loss of this additional layer [47]. How PHB controls the CEM periclinal division is still not known. In *Arabidopsis*, ectopic expression of PHB indirectly promotes *CYCD6;1* expression independently from SHR, therefore PHB might control CEM division acting on this gene. Moreover, whether this mechanism is conserved in other distant relatives with multiple cortical layers or whether this mechanism adds to the one controlled by SHR is still not known. Future research will allow us to uncover the answers to this interesting question. It was shown that SHR and SCR also play a key role in patterning the differences in radial axis anatomy among species. The multi cortical layered species *Oryza sativa* (rice), indeed, maintains the SHR/SCR interaction, but SHR movements are subject to lower restriction, for promoting multiple cortical layers formation [38,84].

The mechanisms governing root axis formation and maintenance in *Arabidopsis* might be valid for most of the species but may not be universal. It was shown that in the root of most of the species, auxin controls cell division, whereas cytokinin controls differentiation. However, in the fern *Azolla filiculoides*, cytokinin promotes cell division, whereas auxin promotes cell differentiation [85]. Analysis of the molecular mechanisms controlling root axes in species other than *Arabidopsis* will permit us to understand how and when these mechanisms arose and diverged. In this optic, utilization of close relatives of *Arabidopsis* might allow us to understand this crucial point.
